# Is maternal SARS-CoV-2 infection in the first trimester associated with congenital heart defects?

**DOI:** 10.3389/fped.2025.1643423

**Published:** 2025-08-18

**Authors:** Athina Samara, Conrado Milani Coutinho, Paul T. Heath, Asma Khalil

**Affiliations:** ^1^ Department of Women’s and Children’s Health, Karolinska Institutet, Stockholm, Sweden; ^2^ Astrid Lindgren Children’s Hospital, Karolinska University Hospital, Stockholm, Sweden; ^3^ FUTURE, Center for Functional Tissue Reconstruction, University of Oslo, Oslo, Norway; ^4^ Hospital das Clínicas, Ribeirão Preto Medical School, University of São Paulo, Ribeirão Preto, Brazil; ^5^ Centre for Neonatal and Paediatric Infection & Vaccine Institute, Institute of Infection and Immunity, City St George’s University of London, London, United Kingdom; ^6^ Fetal Medicine Unit, St George’s University Hospitals NHS Foundation Trust, University of London, London, United Kingdom; ^7^ Vascular Biology Research Centre, Molecular and Clinical Sciences Research Institute, City St George’s University of London, London, United Kingdom; ^8^ Royal College of Obstetricians and Gynaecologists, London, United Kingdom

**Keywords:** SARS-CoV2, congenital heart defects, maternal infection, COVID-19, CHD

## Abstract

Emerging evidence suggests a potential link between maternal SARS-CoV-2 infection during early pregnancy and the development of congenital heart defects (CHD) in offspring. Although vertical transmission of SARS-CoV-2 is rare, the virus has been associated with placental complications and increased maternal morbidity. Recent studies from China report increased rates of CHD and anomalies such as situs inversus when infection occurs during gestational weeks 4–6, a critical window for cardiac development. Additional reports from different parts of the world also highlight post-pandemic increases in specific cardiac anomalies, including ventricular septal defects (VSDs). Multiple mechanisms may underlie these associations. SARS-CoV-2 can induce placental inflammation, compromise the cytotrophoblast barrier, and impair nutrient and gas exchange, potentially leading to fetal hypoxia and disrupted morphogenic signalling. Furthermore, maternal inflammation and elevated cytokines, along with viral effects on ACE2-expressing fetal cardiac progenitors, could further affect proliferation, differentiation, and apoptosis during cardiac development. Co-infections, hormone disruption, and maternal stress could also contribute. There is an urgent need for longitudinal studies with comprehensive maternal-fetal data, including infection timing, vaccine status, and biological sampling. These will be essential to delineate the multifactorial impacts of maternal infection on fetal cardiac development and long-term outcomes. Special focus should be placed on infections during early pregnancy (weeks 4–7), the period of cardiac septation and left-right asymmetry establishment, to determine causality and inform prevention strategies.

## Is maternal SARS-CoV-2 infection in the first trimester associated with congenital heart defects?

Currently, there is limited evidence regarding an association between SARS-CoV-2 infection during pregnancy and the development of congenital anomalies (1). Although reports of vertical transmission to the fetus are rare (2, 3), SARS-CoV-2 is known to increase maternal mortality and morbidity and to cause placental complications, including stillbirth and pre-eclampsia (4–6). Recent data suggesting a link between congenital anomalies and SARS-CoV-2 exposure during early gestation highlight the need for further investigation to clarify the potential risks and mechanisms involved.

In November 2023, Wang et al. reported an increase in the incidence of situs inversus in a large birth cohort following the lifting of pandemic-associated restrictions in China (7). This was further supported by a Chinese case-control study that analysed 52 pregnancies with situs inversus and 208 matched controls between January and October 2023 (8). While no overall association was found between SARS-CoV-2 infection and situs inversus, a significant correlation emerged when infection occurred specifically during gestational weeks 4–6. Infections outside this critical window showed no association, even after adjusting for covariates. Another study also showed that SARS-CoV-2 infection during weeks 5–6 of the first trimester significantly increased the risk of fetal left-right (LR) asymmetry disorders (9). A separate study from China found that newborns of mothers infected with COVID-19 during pregnancy had a significantly higher prevalence of cardiac ultrasound abnormalities (10%) compared to the control group (4%) (10). Most abnormalities were observed in cases where maternal infection occurred before 8 weeks of pregnancy. In addition, the single-centre annual incidence of congenital heart defects (CHD) peaked at 5.5% in 2023, marking a notable increase (10). A summary of the findings of the studies from China is shown in Table 1.

**Table 1:** Summary of the findings regarding maternal SARS-CoV-2 infection and congenital heart defects from studies conducted by researchers in China.

Country	Study period	Study results
China (7)	Compared periods January 1, 2014 through December 31, 2022, and January 1, 2023 through July 31, 2023.	Investigated annual incidence of SI, SIT and SIP (per 10,000 ± SD) from 2014 to 2022: SI 5.6 ± 1.1; SIT 4.0 ± 0.9; SIP 1.5 ± 0.5, compared with Jan.1 to Jul. 31, 2023: SI 23.6; SIT 21.9; SIP 1.7. Fold increase: SI 4.2; SIT 5.4; SIP 1.1
China (10)	January to December 2023. Also investigated annual incidence rates of CHD from 2020 to 2023.	A statistically significant difference in cardiac ultrasound abnormalities prevalence: 10.08% in the maternally infected “COVID-19 group” vs. 4.13% in the uninfected ‘control group (*p* = 0.012). 11 involved maternal infections before 8 weeks of pregnancy, and 1 at 23 weeks. Cardiac abnormalities in COVID-19 group included ASD (8 cases), PFO (6), VSD (2), and PDA (3). In the control group, abnormalities in 17 newborns: ASD (2 cases), PFO (15), VSD (2), and PDA (10). Incidence rates 1.1% in 2020, 2.36% in 2021, and 3.9% in 2022 (*p* < 0.001).
China (9)	LR fetal asymmetry disorders data from 2018 to 2023, evaluating incidence trends. Also, a case-control study (Jan–May 2023) of fetuses with LR asymmetry disorders vs. normal fetuses (1:1 ratio, 14–39 weeks’ gestation).	From 2018 to 2023, LR asymmetry disorder incidence rates were 0.17, 0.63, 0.61, 0.57, 0.59, and 3.24 per 1,000 cases. A case-control study of 30 affected and 30 normal fetuses identified SARS-CoV-2 infection (96.7% vs. 3.3%, *P* = 0.026) and first-trimester infection (96.6% vs. 3.5%, *P* = 0.008) as risk factors, with odds ratios of 10.55 and 13.07, respectively. Most infections (88.1%) occurred at 5–6 weeks gestation. Of fetuses with LR asymmetry disorders, 43.7% had associated malformations, 90.9% of which were cardiac.
China (8)	Between January 1 and October 31, 2023	Enrolled 52 pregnant women diagnosed with fetal SI and 208 matched controls with normal fetuses; fetal SI significantly associated with maternal SARS-CoV-2 infection in gestational weeks 4–6 [(aOR) 6.54 (95% confidence interval 1.76–24.34)], but not at other gestational ages.

LR, left-right; SI, situs inversus; SIT, situs inversus totalis with dextrocardia; SIP, partial situs inversus with levocardia; ASD, atrial septal defect, PFO, patent foramen ovale, VSD, ventricular septal defect; PDA, patent ductus arteriosus.

A single-centre US study investigated prenatal diagnosis rates of newborns with critical congenital heart defects (CCHDs) admitted for cardiac intervention during the COVID-19 pandemic (March 2020 to March 2021) and compared them with pre-pandemic periods (2009–2012 and 2013–2016). This study found that during the pandemic, patients had a 2.17 times higher odds of receiving a prenatal diagnosis of CCHD compared to the pre-pandemic period (11). Subsequent to this, a larger US-based study analysing data from the Centers for Disease Control and Prevention (CDC) demonstrated an increase in cyanotic congenital heart anomalies during the COVID-19 pandemic, though this study did not include analysis of CCHD subtypes, maternal SARS-CoV-2 infection, stillbirths, or pregnancy termination (12).

Unfortunately, analyses of the impact of COVID-19 maternal infections on CHD and other anomalies from other countries is limited, with further research pending. In Canada, pandemic-relevant congenital anomaly prevalence data is not available after 2020 (13). In Europe, the Joint Research Centre (JRC) technical report on European Surveillance of Congenital Anomalies (EUROCAT) presented the results of statistical monitoring for pan-European trends across 94 anomaly subgroups for 2012–2021 (14). This found that the incidence of patent ductus arteriosus (PDA) as an isolated CHD in term infants decreased significantly over the study period. However, this analysis did not include all European countries due to incomplete registry coverage. Two studies outside the registry have demonstrated an increase in CHD. In Turkey, a retrospective cohort study of 254 infants identified that preterm infants weighing up to 1.5 kg with maternal SARS-CoV-2 infection had a significantly higher rate of PDA (38% vs. 15%) (15). Similarly, a study in Ukraine found that 13.8% of fetuses of mothers infected with SARS-CoV-2 during pregnancy had congenital anomalies, with cardiovascular malformations second in prevalence only to facial anomalies (16).

**Figure 1:**
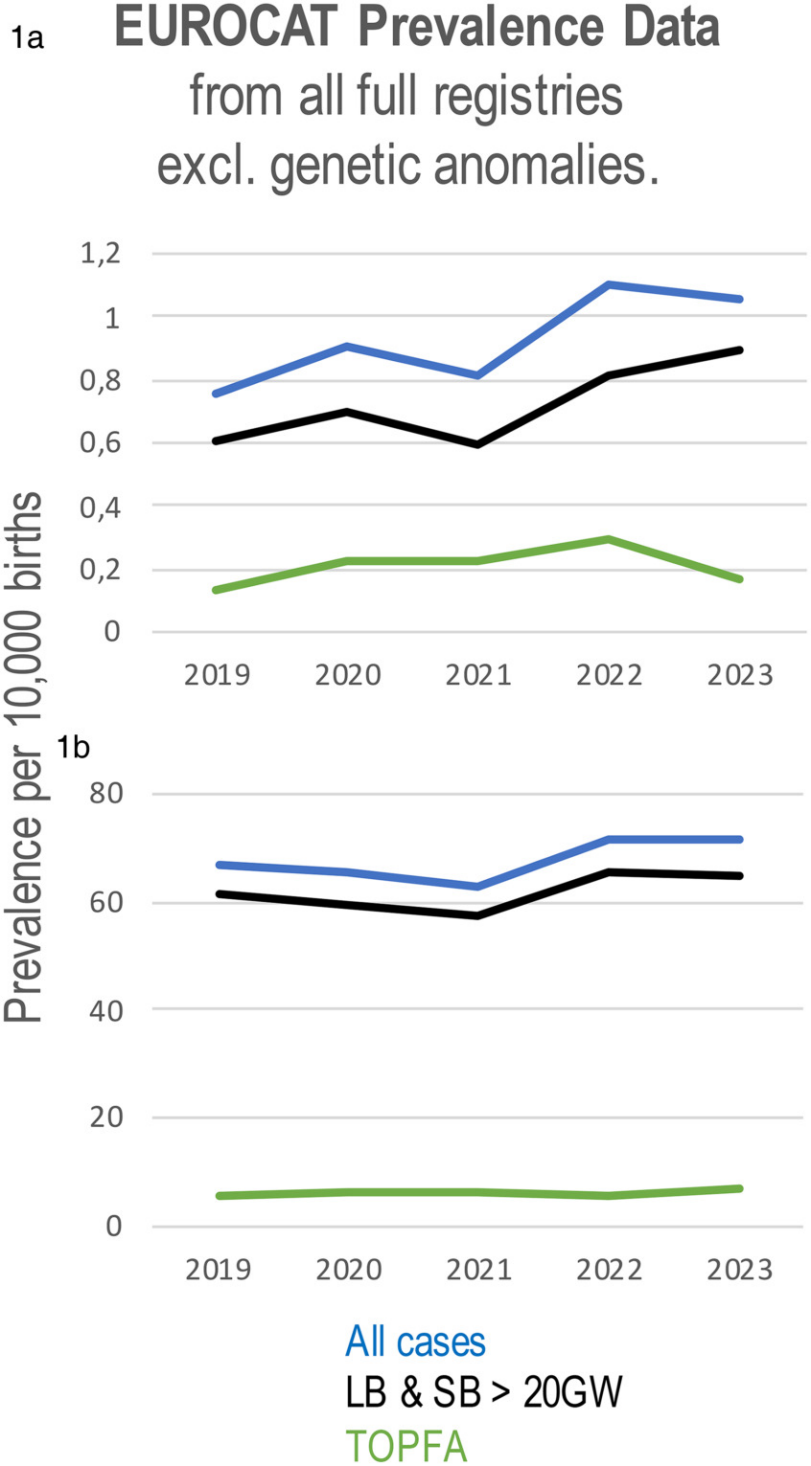
Prevalence of situs inversus **(a)** and all congenital heart defects **(b)** per 10,000 births using data from all full registries of EUROCAT from 2019 to 2023. CHD prevalence includes live births and stillbirths from 20 weeks’ gestation (LB & SB >20GW), cases of termination of pregnancy due to fetal anomaly (TOPFA), and all cases combined.

The EUROCAT data from 2019 to 2023 has identified a rise in the incidence of situs inversus (Figure 1a); while there was a decline in live births and stillbirths with the condition in 2021, there was an increase in pregnancy terminations for situs inversus during the same period and an increase in the incidence of situs inversus between 2021 and 2023. Prevalence data analysed by EUROCAT demonstrated a slight decline in the incidence of CHD from 65.75 cases per 10,000 births in 2020 to 63.01 in 2021, followed by an increase to 71.63 cases per 10,000 births in 2022 and 71.47 in 2023. This trend reflects a rebound after the initial COVID-19 pandemic period, with overall prevalence remaining higher than in 2020 and 2021. (Figure 1b) (17). The EUROCAT data also show an increase in the prevalence of VSDs from 32.42 in 2019 to 37.39 in 2022, further increased to 39.5 in 2023, while the prevalence of tetralogy and pentalogy of Fallot cases decreased from 2019 to 2022, accompanied by an increase in 2023.

Additionally, surveillance data from the Argentinian National Registry of Congenital Anomalies (Registro Nacional de Anomalías Congénitas, RENAC), report an upward trend in cases of tetralogy and pentalogy of Fallot, with rates increasing from 2.12 (95% CI: 1.60–2.76) in 2019 to 2.41 (1.83–3.11) in 2020, 2.70 (2.08–3.46) in 2021, 2.16 (1.61–2.84) in 2022, and 2.84 (2.17–3.66) in 2023 (18).

**Figure 2:**
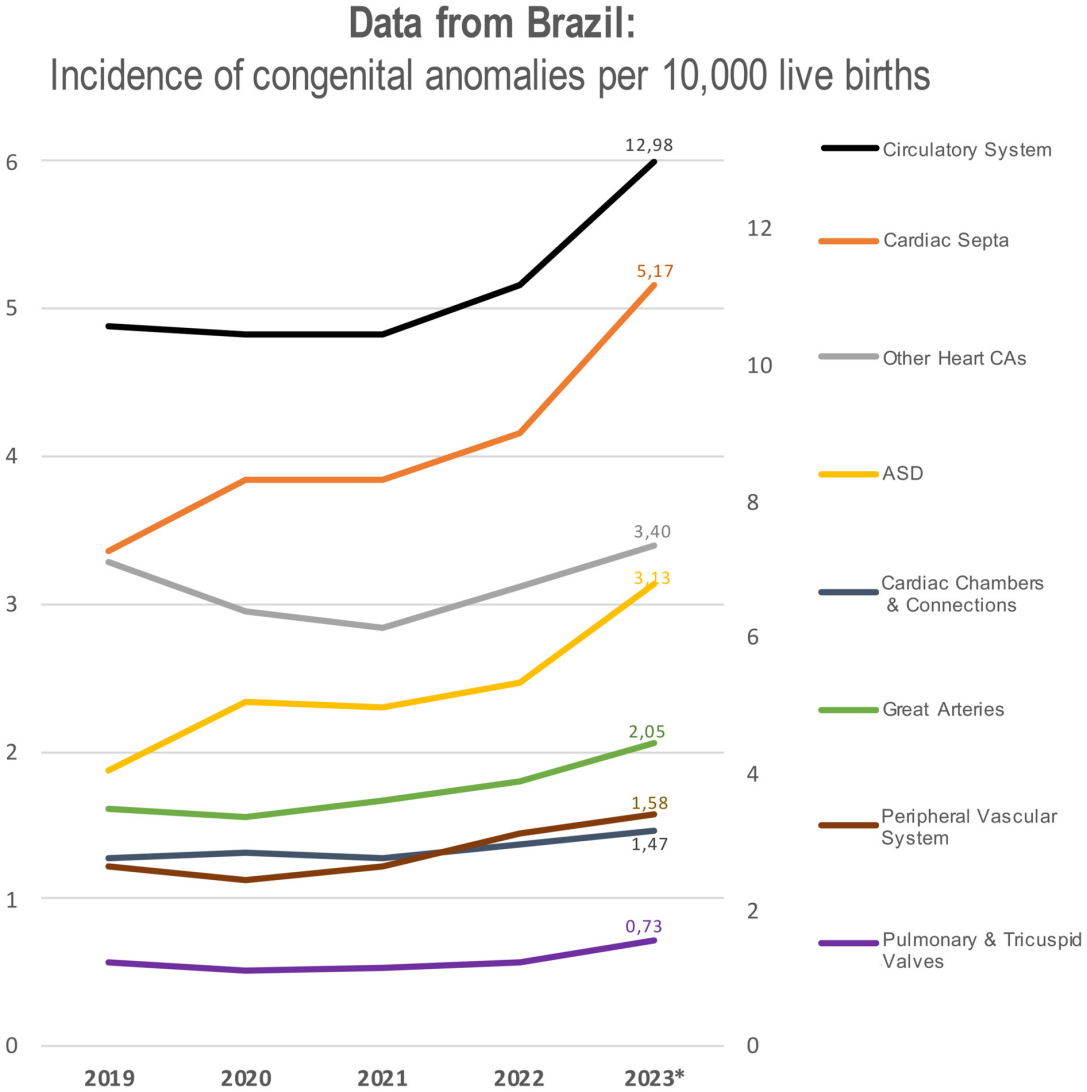
Annual incidence of congenital malformations of the circulatory system per 10,000 live births in Brazil from 2019 to 2023. Categories include specific CHD types: defects of cardiac septa, chambers and connections, great arteries, pulmonary/tricuspid valves, and peripheral vascular system, classified according to ICD-10 Q20–Q28. The graph has two axes: the right axis specifically refers to circulatory system malformation, while the left axis represents the incidence of all other congenital malformations.

We have examined Brazil's publicly available data from 2019 to 2023 to calculate the annual incidence of selected congenital anomalies per 10,000 live births (19). Starting from the pandemic years, there has been an increase in reported cases of congenital malformations of the circulatory system (Q20–Q28) (20), including defects in cardiac septa (Q21), the great arteries (Q25), cardiac chambers and connections (Q20), pulmonary and tricuspid valves (Q22), the peripheral vascular system (Q27), and other heart malformations (Figure 2). Of note, the incidence of malformations in cardiac septa steadily increased from 3.36 in 2019 to 5.17 in 2023 (19). Similarly, the data revealed a rising incidence in ASDs, which continues to increase through 2023 (19).

As reflected in the findings discussed above, the impact of SARS-CoV-2 infection in early pregnancy may be associated with a statistically significant increase in CHD. It should be emphasised that COVID-19 vaccination in early pregnancy does not appear to be associated with a risk of congenital anomalies (21).

All these reports from China (7–10), Europe (14, 17), the US (11, 12), Brazil (19), Argentina (18), Turkey (15) and Ukraine (16), indicate a post-pandemic rise in specific CHDs, highlighting a critical gap in our understanding of the effects of early maternal SARS-CoV-2 infection on fetal development. These effects may be explained by interconnected disruptions affecting fetal heart development, particularly during the critical septation period between weeks 4 and 7 of gestation (22), with maternal first trimester infections being linked to CHD (23). Secondary factors like co-infections [e.g., cytomegalovirus (CMV)] (23, 24) and maternal inflammation, including elevated cytokines, can also disrupt cardiac morphogenesis pathways (25, 26). Furthermore, SARS-CoV-2 induced placental inflammation may weaken the cytotrophoblast layer, compromising the placental barrier, nutrient, and gas exchange (27, 28). This disruption, combined with maternal hypoxia from respiratory distress or vascular damage, may affect cardiac development, especially the ventricular septum and outflow tracts (29–31). The compromised cytotrophoblast barrier may also allow increased fetal exposure to maternal inflammatory cytokines (28) and pathogens, disrupting signalling pathways crucial for cardiac morphogenesis, such as Wnt, BMP, and Notch (29, 32).

Infection-induced loss of placental barrier integrity also affects hormone production (e.g., hCG) (33), impacting angiogenesis and myocardial development. Additionally, the virus may directly affect fetal cells via ACE2 expression in cardiac progenitors, disrupting proliferation, differentiation, and apoptosis (28). Maternal stress, elevated cortisol, and epigenetic changes (e.g., DNA methylation) could further complicate heart development. While direct animal studies linking COVID-19 to CHD are currently unavailable, related data demonstrate placental dysfunction, fetal growth restriction, and neurodevelopmental delays (34). This evidence supports a plausible mechanistic pathway worth exploring in animal models focused on cardiac outcomes. These complex factors (summarized in Table 2) highlight the need for further research into the impacts of maternal SARS-CoV-2 infection on fetal heart development.

**Table 2:** Examples of effects of maternal SARS-CoV-2 infection on fetal heart development.

Category	Primary features	Specific details
Disruptions during critical septation period (weeks 4–7)	Interconnected disruptions affecting fetal heart development	Impaired ventricular septum & outflow tract formation
Maternal infections & secondary factors	First trimester infections associated with CHD	Examples include co-infections (e.g., cytomegalovirus)
Maternal hypoxia & placental insufficiency	Maternal respiratory distress, vascular damage	Impaired cardiac development, particularly ventricular septum & outflow tracts
Maternal inflammation & immune responses	Elevated cytokines disrupting signalling pathways	Molecular pathways affected (including Wnt, BMP, Notch)
Placental inflammation & barrier compromise	Weakening of cytotrophoblast layer	Increased permeability, affecting nutrient & gas exchange, impairing differentiation
Syncytiotrophoblast dysfunction	Reduced production of essential hormones	Compromised angiogenesis & myocardial tissue development
Fetal exposure & signalling disruption	Increased fetal exposure to maternal inflammatory cytokines & pathogens	Disruption of cardiac morphogenesis pathways (e.g., Wnt, BMP, Notch)
Direct viral impacts on the fetus	ACE2 expression in cardiac progenitor cells	Interference with proliferation, differentiation, apoptosis, & retinoic acid pathways
Epigenetic & Hormonal changes	Infection-related stress & inflammation	DNA changes altering molecular pathways; maternal stress elevating cortisol

The table highlights factors contributing to disruptions in fetal heart development during the critical septation period (weeks 4–7). Maternal first trimester infections are linked to congenital heart defects (CHD), with co-infections potentially worsening these effects. Maternal inflammation can disrupt cardiac morphogenesis pathways and SARS-CoV-2-induced placental inflammation may weaken the cytotrophoblast layer, compromising the placental barrier, increasing permeability, and interfering with nutrient and gas exchange, critical for early fetal development. The loss of barrier integrity and impaired cytotrophoblast differentiation can further compromise fetal heart development during the early gestational weeks. Furthermore, syncytiotrophoblast dysfunction may reduce essential hormone production, impairing angiogenesis and myocardial development. Maternal hypoxia, from respiratory distress or placental insufficiency, may further affect cardiac development, especially the ventricular septum and outflow tracts. Infection-related maternal stress and inflammation may cause epigenetic changes and hormonal imbalances, further increasing developmental risks. Notably, maternal SARS-CoV-2 infection may directly impact the fetus through ACE2 receptors in cardiac progenitor cells, disrupting cell proliferation, differentiation, apoptosis, and other pathways crucial for heart tube formation.

To understand the effects of early maternal SARS-CoV-2 infection on fetal development, comprehensive and detailed data collection is now essential. This should include demographic information such as age, ethnicity, socioeconomic status, and pre-existing health conditions, enhanced by environmental and lifestyle factors like stress, diet, and exercise should be considered, as these can also impact outcomes, as these factors may influence maternal and fetal health outcomes.

Clinical data should track the severity, timing and SARS-CoV-2 strain of maternal infection, including comorbidities, hospitalisation, and medication use, alongside fetal health indicators like growth measurements, ultrasounds, and any congenital abnormalities. Additionally, information on maternal vaccination status, type of vaccine, timing relative to pregnancy, and booster doses, are also important. Insight into the maternal immune response, such as viral load, circulating antibody and cytokine levels, can elaborate on the biological effects of the infection or vaccination.

To better understand the effects of early maternal SARS-CoV-2 infection on fetal development, several types of samples should be collected across multiple time points during pregnancy. Maternal blood samples are essential to explore the DNA methylation patterns and mRNA abundance, providing insight into how maternal infection may alter gene expression, focusing on the immune response and fetal development genes. Amniotic fluid or cord blood samples can be analysed for fetal DNA, to explore biomarkers and epigenetic changes linked to the maternal condition, in addition to inflammatory cytokines and viral load, helping to understand the direct impact of the infection to the fetus. Placental tissue, collected at birth, would allow for the assessment of epigenetic landscape modifications that affect fetal development. Imaging data, such as regular ultrasounds, should also be collected to monitor fetal growth, development, and any abnormalities. By collecting these samples, we can track how maternal infection and vaccination, along with other factors like stress and medication, could influence fetal congenital heart defect formation. We can also explore genetic and environmental interactions that may contribute to long-term effects. In addition, future studies stratifying risk should also explore fetal genetic conditions, such as trisomy 21 (Down syndrome), in which approximately 40%–50% of individuals present with CHD (35).

We must continue to collect and analyse data on the development of CHD following maternal SARS-CoV-2 infection and conduct detailed studies to determine how maternal infection may influence the development of CHD and other anomalies. Areas of particular importance for future research are infection during the critical 4–7-week period in early pregnancy, and whether SARS-CoV-2 contributes to left-right asymmetry, septal development and lateral visceralisation disruption, all of which have significant implications for maternal and fetal health.

## Data Availability

The original contributions presented in the study are included in the article/Supplementary Material, further inquiries can be directed to the corresponding author.
